# Bilateral neuroinflammatory processes in visual pathways induced by unilateral ocular hypertension in the rat

**DOI:** 10.1186/s12974-016-0509-7

**Published:** 2016-02-20

**Authors:** Anaïs Sapienza, Anne-Laure Raveu, Elodie Reboussin, Christophe Roubeix, Céline Boucher, Julie Dégardin, David Godefroy, William Rostène, Annabelle Reaux-Le Goazigo, Christophe Baudouin, Stéphane Melik Parsadaniantz

**Affiliations:** Sorbonne Universités, UPMC University of Paris 06, Institut de la Vision, 17 rue Moreau, 75012 Paris, France; INSERM U968, Institut de la Vision, 17 rue Moreau, 75012 Paris, France; CNRS UMR_7210, Institut de la Vision, 17 rue Moreau, 75012 Paris, France; CHNO des Quinze-Vingts, DHU Sight Restore, INSERM-DHOS CIC, 28 rue de Charenton, 75012 Paris, France; Department Ophthalmology, Hopital Ambroise Pare, AP HP, F-92100 Boulogne, France; University Versailles St Quentin En Yvelines, F-78180 Montigny-Le-Bretonneux, France

**Keywords:** Ocular hypertension, Neuronal degeneration, Neuroinflammation, Tissue macrophage activation, Superior colliculi, Visual deficiency

## Abstract

**Background:**

Glaucoma is one of the leading causes of irreversible blindness in the world. The major risk factor is elevated intraocular pressure (IOP) leading to progressive retinal ganglion cell (RGC) death from the optic nerve (ON) to visual pathways in the brain. Glaucoma has been reported to share mechanisms with neurodegenerative disorders. We therefore hypothesize that neuroinflammatory mechanisms in central visual pathways may contribute to the spread of glaucoma disease. The aim of the present study was to analyze the neuroinflammation processes that occur from the pathological retina to the superior colliculi (SCs) in a rat model of unilateral ocular hypertension induced by episcleral vein cauterization (EVC).

**Results:**

Six weeks after unilateral (right eye) EVC in male Long-Evans rats, we evaluated both the neurodegenerative process and the neuroinflammatory state in visual pathway tissues. RGCs immunolabeled (Brn3a^+^) in ipsilateral whole flat-mounted retina demonstrated peripheral RGC loss associated with tissue macrophage/microglia activation (CD68^+^). Gene expression analysis of hypertensive and normotensive retinas revealed a significant increase of pro-inflammatory genes such as CCL2, IL-1β, and Nox2 mRNA expression compared to naïve eyes. Importantly, we found an upregulation of pro-inflammatory markers such as IL-1β and TNFα and astrocyte and tissue macrophage/microglia activation in hypertensive and normotensive RGC projection sites in the SCs compared to a naïve SC. To understand how neuroinflammation in the hypertensive retina is sufficient to damage both right and left SCs and the normotensive retina, we used an inflammatory model consisting in an unilateral stereotaxic injection of TNFα (25 ng/μl) in the right SC of naïve rats. Two weeks after TNFα injection, using an optomotor test, we observed that rats had visual deficiency in both eyes. Furthermore, both SCs showed an upregulation of genes and proteins for astrocytes, microglia, and pro-inflammatory cytokines, notably IL-1β. In addition, both retinas exhibited a significant increase of inflammatory markers compared to a naïve retina.

**Conclusions:**

All these data evidence the complex role played by the SCs in the propagation of neuroinflammatory events induced by unilateral ocular hypertension and provide a new insight into the spread of neurodegenerative diseases such as glaucoma.

## Background

Glaucoma is the second leading cause of irreversible blindness and is projected to affect 80 million people worldwide by 2020, including at least 7 million people who will become bilaterally blind [1]. Glaucoma is a chronic degenerative optic neuropathy in which intraocular pressure (IOP) is abnormally elevated, leading to the progressive loss of retinal ganglion cells (RGCs) and an alteration of their axons. Today, high IOP is the major risk factor and the current treatment strategy focuses on its ability to reduce IOP by pharmacological or surgical means [2]. However, other mechanisms than IOP increase seem to be involved in the development and progression of this degenerative disease. Indeed, glaucoma progression can be observed in 15–25 % of patients despite appropriate IOP control [3, 4], and normal-tension glaucoma comprises a significant proportion of glaucoma cases in which an elevated IOP cannot explain neurodegeneration. In addition, abnormal activation of autoimmunity has been observed, which could induce the loss of RGCs in glaucoma patients without elevated IOP [5]. Consequently, this disease may progress through an IOP-independent mechanism and may involve other factors. In this way, numerous data indicate that inflammatory responses in the retina could appear earlier than the IOP rise [6–9]. In DBA/2J mouse glaucoma model, it has been reported that young mice present microglial activation in their retina and optic nerve in absence of an elevated IOP context [10, 11]. In addition, it has been suggested that astrocytes and Müller cells exhibit an activated phenotype with morphologic changes in retina before the increase of IOP. However, inflammatory events induced by glaucoma occur not only in the retina and the optic nerve (ON). In the human brain, it has been clearly observed that neurodegenerative processes also affect all layers of the lateral geniculate nucleus (LGN), which constitutes the major target of RGCs (corresponding to up to 80–90 % of RGC fibers) [12, 13]. In contrast to humans, in the rodent visual system, after decussating, the majority of RGC axons (90 %) project directly to the contralateral superior colliculus (SC) [14–16]. Thus, in a rat model of acute intraocular hypertension, a strong astrogliosis has been shown in the SC contralateral to the hypertensive eye [17]. Moreover, it has been recently demonstrated that a deregulation of cytokine signaling in the SC can appear before the IOP or axonal degeneration in the DBA/2J mouse glaucoma model [18].

The normotensive eye could also be affected by these inflammatory changes. It has been observed that microglia and astrocyte reactivity is upregulated in the retina contralateral to the hypertensive eye in mice [19, 20]. However, the pathophysiology of neuroinflammatory events, responsible for contralateral eye alteration, remains to be clarified. Nevertheless, some data have provided evidence of RGC axon projection from one retina to the other via the optic chiasm and called retino-retinal projection pathway [21, 22]. However, this bypass pathway remains minor and concerns only a marginal proportion of axons [23, 24]. The SC could be the major structure of communication by which neuroinflammatory events could spread to both eyes. In rodents, after the optic chiasma, each SC receives RGC axonal projections from both retinas in a proportion of around 85/15 % [14–16, 22, 23, 25].

The purpose of this study was to understand how neuroinflammatory processes could spread to the normotensive eye in the unilateral ocular hypertension glaucoma model. To respond to this question, we first analyzed the neuroinflammatory consequences of unilateral ocular hypertension in visual pathway tissues including retinas, optic nerves, and SCs in both episcleral vein cauterization (EVC) and contralateral tissues. Then to demonstrate the role played by the SC in the propagation of neuroinflammatory events induced by glaucoma, we used a model of unilateral injection of TNFα into the right SC.

## Methods

### Animal model

Fifty male 8-week-old Long-Evans rats (Janvier Labs) weighing 250–300 g were used. Animals were kept in pathogen-free conditions with food and water available ad libitum and housed in a 12-h light/12-h dark cycle.

All experiments were conducted after evaluation and approval by the Institutional Animal Care and Use Committee, Comité d'éthique pour l’expérimentation animale Charles Darwin (reference number: 03858.02), in accordance with the guidelines from Directive 2010/63/EU of the European Parliament on the protection of animals used for scientific purposes. For sample size and number of rats used in each experiment see Table 1.

**Table 1:** Sample size and number of rats used in each experiment

Episcleral vein cauterization experiments	RT-PCR	*n* = 8–10 rats	2	20
	Immunohistofluorescence	*n* = 6–7 rats	4	28
Stereotaxic CTB-Alexa 594 injection experiments	Histofluorescence	*n* = 4 rats	1	4
Stereotaxic TNFα injection experiments	RT-PCR	*n* = 8–10 rats	2	20
	Immunohistofluorescence	*n* = 6 rats	2	12
	Total number of rats			84

### Induction of ocular hypertension and IOP measurements

The surgical ocular hypertension (OHT) model was induced in the right eye of each rat by cauterization of three episcleral veins after conjunctival dissection under general anesthesia (intraperitoneal injection of ketamine (100 mg/kg, Virbac, Vauvert, France) and xylazine (10 mg/kg, Bayer HealthCare, Whippany, USA) as previously described [26, 27]. Briefly, the superotemporal, superonasal, and inferotemporal episcleral veins were located and cauterized using a standard disposable ophthalmic cautery. The left eyes were used as contralateral controls, and eyes from naïve animals were used as naïve controls. At baseline and after the surgery, the animals were maintained for a 6-week period and monitored for IOP once a week using a handheld tonometer (TonoLab, Medtronics, Jacksonville, USA). In this study, IOP average values were calculated from three initial values from eight rats for the three groups (hypertensive, normotensive, and control eyes) at each time point. During IOP measurements, all the animals were awake.

### Stereotaxic CTB-Alexa 594 and TNFα injection

Fourteen male Long-Evans rats (7 weeks old) were deeply anesthetized with ketamine (100 mg/kg) and xylazine (10 mg/kg) and placed on a digital stereotaxic frame. A small drop of ophthalmic gel was placed on both eyes to prevent corneal dehydration throughout the procedure. The scalp was removed, and the skull was exposed. A small craniotomy (1 mm × 1 mm^2^) was drilled above the SC in the right hemisphere (mediolateral, 1.6 mm; anteroposterior; −6.72 mm; dorsoventral, −4.2 mm from the bregma). Four microliters of sterile recombinant rat TNFα (25 ng/μl) (Biorad Laboratories, Nazareth, Belgium) or cholera toxin subunit B (1 μg/μl) Alexa 594 (Life Technologies, Paisley, UK) were unilaterally injected into the SC (1.2 μl/min). After injection, the needle was left for 5 min and removed slowly. The skull skin was stitched and to recover, rats were placed for 1 h at 30 °C. The tissue was analyzed 2 weeks after TNFα injection and 5 days after CTB injection.

### Optomotor response

The optokinetic tracking threshold was measured, under photopic conditions, by observing the optomotor responses of rats to rotating sinusoidal gratings (OptoMotry, CerebralMechanics, Alberta, Canada). Rats reflexively respond to rotating vertical gratings by moving their head in the direction of the grating rotation [28]. The protocol yields independent measures of right and left eye acuity based on the unequal sensitivity of the two eyes to pattern rotation: the right and left eyes are most sensitive to counter-clockwise and clockwise rotations, respectively. Once the rat became accustomed to the pedestal, the test was initiated by presenting the rat with a sinusoidal striped pattern that rotates either clockwise or counter-clockwise and varied widths. The software randomly increased spatial frequency of the grating until the animal no longer responded. The process of changing the spatial frequency of the test grating was repeated a few times until the highest spatial frequency the rat could track was identified, which defines the optokinetic tracking threshold under the experimental conditions. Experiments were conducted after TNFα administration by two observers masked to the animal’s treatment and previously recorded thresholds.

### Immunohistochemistry

#### Tissue preparation

At the end of the experiment, rats from each group were deeply anesthetized via a single IP injection of ketamine (75 mg/kg) and xylazine (10 mg/kg) and fixed by a 50 ml intraaortic perfusion of 0.9 % NaCl solution then 400 ml of 4 % paraformaldehyde in 1× phosphate buffer saline (PBS; pH 7.4). After fixation, the eyes were carefully removed and post-fixed for 1 h, then rinsed in 1× PBS solution. The optic nerves were cut right after the eye, and then the brain was removed with optic nerves. The ONs and brains were post-fixed for 24 h and only ONs were cryoprotected for 24 h by immersion in a 1× PBS solution containing 30 % sucrose at 4 °C. For the immunofluorescence experiments, we used optic nerve part the most close to the eye on 3 ml of length. Then the ONs were included in a 7.5 % gelatin, 10 % sucrose mix, and frozen at −20 °C. Retinal tissues were dissected intact from the globe and flat-mounted.

#### Dual immunofluorescence labeling in whole flat-mounted retina

For primary antibody and secondary references and dilutions see Table 2.

**Table 2:** Primary and secondary antibody references and dilutions

	Dilution	Host	Antibody type	Supplier
Primary antibodies				
Brn-3a	1/100	Mouse	Monoclonal	Merck Millipore
Iba1	1/400	Goat	Polyclonal	abcam
CD68	1/200 or 1/400	Mouse	Monoclonal	AbD Serotec
GFAP	1/500	Mouse	Monoclonal	Sigma–Aldrich
CCL2	1/500	Rabbit	Polyclonal	Torrey–Pines
c-fos	1/500	Rabbit	Polyclonal	Santa Cruz
NeuN	1/500	Mouse	Monoclonal	Merck Millipore
p-p38	1/200	Rabbit	Polyclonal	Cell Signaling Technology
Secondary antibodies				
Anti-mouse	1/500	Donkey	Alexa Fluor 594	Life Technologies
Anti-goat	1/500	Donkey	Alexa Fluor 594	Life Technologies
Anti-mouse	1/500	Donkey	Alexa Fluor 488	Life Technologies
Anti-rabbit	1/500	Horse	Biotinylated	Vector Labs
Anti-mouse	1/500	Goat	Biotinylated	Vector Labs
Anti-goat	1/500	Horse	Biotinylated	Vector Labs

The retinas were incubated for 2 h in a blocking-permeabilizing solution of 1× PBS containing 10 % bovine serum albumin (BSA), Triton X-100 2 %, and Tween20 0.5 %. The retinas were incubated for 4 days at 4 °C with monoclonal mouse anti-Brn-3a (1/100, Merck Millipore, Darmstadt, Germany), polyclonal goat anti-Iba1 (1/400, Abcam, Cambridge, UK), or monoclonal mouse anti-CD68 (1/400, AbD Serotec, Oxford, UK), washed with 1× PBS six times for 20 min and incubated with donkey anti-mouse conjugated with Alexa Fluor 594 (Life Technologies) as the secondary antibody. The nuclei were stained with Dapi (1/2000) for 1 h, and the retinal sections were mounted with Fluoromount (Sigma–Aldrich, St. Louis, MO).

#### Dual immunofluorescence labeling for retinas, ON and brain sections

Free-floating brain sections (thickness, 30 μm) were cut with a vibratome (Leica, VT1000S) and collected in 1× PBS. The retina and ON sections (thickness, 10 μm) were cut with a cryostat (Leica, CM3050S) and kept at −20 °C. The sections were washed several times in 1× PBS, blocked and permeabilized with 4 % BSA, 4 % normal horse serum (NHS), and 0.3 % Triton X-100 in 1× PBS for 2 h.

Sections were subsequently incubated overnight at 4 °C in 1× PBS containing 2 % BSA, 2 % NHS, and 0.15 % Triton X-100 with the following primary antibodies: polyclonal goat anti-Iba1 (1/400, Abcam), monoclonal mouse anti-GFAP (Glial fibrillary acidic protein) (1/500, Sigma–Aldrich), monoclonal mouse anti-CD68 (1/200, Abd Serotec), polyclonal rabbit anti-CCL2 (1/500, Torrey-Pines), polyclonal rabbit anti-c-fos (1/500, Santa Cruz, CA), monoclonal mouse anti-NeuN (1/500, Merck Millipore), and rabbit anti-p-p38 (1/200, Cell Signaling Technology, Beverly, MA).

Secondary antibodies included donkey anti-mouse and anti-goat antibodies conjugated with Alexa Fluor 488 and 594 (1/500, Life Technologies) and biotinylated horse anti-rabbit antibodies (1/500, Vector Labs, Burlingame, USA) followed by a streptavidin Alexa Fluor 488 and 594 conjugate (1/500, Life Technologies). Staining with Dapi (1/2000), a nuclear marker was also used. Sections were then washed, mounted on gelatin-coated glass slides, and coverslipped with Fluoromount. Control sections were processed in parallel in the absence of either primary or secondary antibodies.

#### Immunolabeling

Free-floating sections were incubated with 3 % H_2_O_2_ for 20 min and then with blocking/permeabilizing buffer (see previous section). Sections were incubated overnight at 4 °C (in 1× PBS containing 2 % BSA, 2 % NHS and 0.15 % Triton X-100) with GFAP and Iba1 antibodies. The sections were incubated with biotinylated goat anti-mouse and horse anti-goat antibodies (1/500, Vector Labs) for 1 h and the Vectastain ABC kit (1/250, Vector Labs) for 1 h. The color reaction was developed for 3–5 min with DAB^+^ (Sigma–Aldrich). The sections were washed in 0.05 Tris buffer, pH 7.6, dehydrated in Safesolv (no toxic xylene substitute, VWR Q Path, Leighton Buzzard, UK) and coverslipped with Eukitt mounting media.

#### Data analysis

Images were captured with a DM6000 microscope (Leica, Nanterre, France) and analyzed using MetaMorph software (Molecular Devices, Sunnyvale, CA). For labeling density analysis, image files were inverted and opened in gray-scale. Subsequently, using the thresholding function of Fiji [29] to discriminate objects of interest from the surrounding background, the total surface occupied by immunoreactive structures (i.e., total stained pixels) above this set threshold was estimated within a standard area (ROI manager). Images were taken at objective ×20 on a Leica microscope. The resulting values were expressed in surface units corresponding to 306,804 μm^2^. The results were expressed as the mean ± SEM of six to eight values per structure and per animal.

Images for co-localization were taken with an Olympus FV1000 laser-scanning confocal microscope (Olympus, Philadelphia, USA) and acquisitions were obtained using the Olympus Fluoview software version 4.1.

### RGCs and activated tissue macrophage/microglia labeling and counting

Eight microscopic images were captured using a ×20 objective in the peripheral retina and then peripheral, middle, and central regions in whole flat-mounted retina labeled with Brn-3a and CD68 (which plays a role in phagocytic activities of tissue macrophages) antibodies, respectively. Images were captured with a DM6000 microscope (Leica), and automatic enumeration of RGC nuclei was assessed in a blind manner with MetaMorph software (MolecularDevices). Iba1- and CD68-positive cells were counted with the Fiji cell-counter plugin (ImageJ software, NIH, Bethesda, MD, USA) [30]. The Iba1- and CD68-positive cells in the central retina were quantified around the ON head. In order to analyze the density of neuronal cell population (labeled with the NeuN marker) in SC of EVC and control groups we used an automatic counting cell software (Explora Nova Morpho Strider; 2D Analysis).

### mRNA extraction, reverse transcription, and real-time polymerase chain reaction (RT-PCR)

#### Tissue preparation

At the end of the experiment, rats from each group were deeply anesthetized using a single IP injection of ketamine (75 mg/kg) and xylazine (10 mg/kg) and flushed with an intraaortic perfusion of 0.9 % NaCl. The eyes were carefully removed and dissected, and the retinas were frozen in dry ice. The rat brains were detached from the cranial box and directly frozen in carbonic ice. Then the brains were put in stainless steel coronal brain matrix (Harvard Apparatus), and 1-mm slices were taken and micro-dissected. Regions of interest (superior colliculi) were frozen in liquid nitrogen.

#### mRNA extraction, reverse transcription, and RT-PCR

Total RNA levels in retinal tissues and brain punches were assessed using the NucleoSpin RNA II and NucleoSpin RNA XS Purification kit, respectively (Macherey-Nagel, Düren, Germany). RNA concentration was evaluated from absorbance measurements with NanoDrop (ND-1000 spectrophotometer, Wilmington, USA).

#### RT-PCR

First-strand cDNA synthesis (20 μl reaction) was performed with a High-Capacity cDNA Reverse Transcription kit (Applied Biosystems, Life Technologies). Concentrations of each sample were adjusted to 5 ng/μl of cDNA. Real-time PCR amplification of each sample was performed in triplicate, on the 7300 Real-Time PCR system (Applied Biosystems, Life Technologies). TaqMan® Gene Expression Assays (Applied Biosystems) were used for target genes: GFAP (Rn01460868_m1), IL1β (Rn00580432_m1), CCL2 (Rn00580555_m1), Nox2 (Rn00576710_m1), Nox4 (Rn00585380_m1), TNFα (Rn01525859_g1), CD68 (Rn01495634_g1), and glyceraldehyde-3-phosphate dehydrogenase (GAPDH) (Rn01775763_g1). Specific mRNA levels were calculated after normalization of the results for each sample with those for GADPH mRNA. The data are presented as relative mRNA units with respect to control values.

### Statistical analysis

All values are expressed as means ± SEM.

IOP measurement was analyzed using two-way ANOVA for repeated measures followed by the Bonferroni post hoc test. RGC counting, RT-qPCR data, CD68-positive cells, and c-fos counting were analyzed using one-way ANOVA, followed by the Tukey or Kruskal–Wallis or Dunnett multiple comparisons post hoc tests. ON and SC immunostaining levels of GFAP and Iba1 were analyzed using one-way ANOVA followed by the Bonferroni post hoc test. Statistical analyses were performed at the significance level of 0.05 with Prism 6 GraphPad software (San Diego, USA).

## Results

### Ocular hypertension leads to RGC death in the cauterized eye and also affects the contralateral eye

**Fig. 1:**
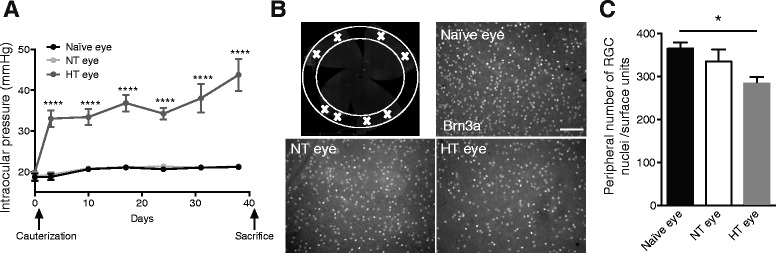
Cauterization leads to an elevated IOP and RGCs death. **a** IOP monitoring of HT right eye, NT eye, and naïve eye over 40 days (*n* = 8 per group). **b** Peripheral area of whole flat-mounted retina marked with a *white cross* matching the region of interest. Whole flat-mounted retina immunolabeled with Brn3a antibody. Representative images of the peripheral area showing immunopositive staining of RGCs in a naïve eye, NT eye, and HT eye. Scale bar = 100 μm. **c** Quantification of peripheral RGC density per retina using automated nuclei counting in a naïve (*n* = 7), NT (*n* = 6), and HT eye (*n* = 6). Each density corresponds to an average of RGCs determined from eight peripheral images per retina. Data are expressed as means ± SEM. Two-way ANOVA for repeated measures followed by Bonferroni post hoc test was used for IOP: ****p < 0.0001 naïve and NT versus HT eyes. One-way ANOVA, followed by the Dunnett multiple comparisons test was used for RGC counting: *p < 0.05 naïve versus HT eyes

IOP was measured with a tonometer and followed once a week for 40 days. Immediately after the surgery, the cauterized animals showed elevated IOP, which was stable between 30 and 40 mmHg. Naïve and normotensive (NT) eyes showed sustained normal IOP at 20 mmHg (Fig. 1a). Six weeks after cauterization, the animals were sacrificed and whole flat-mounted retina immunolabeling of RGCs was performed. All experiments were done at this time point. The images represent peripheral areas of naïve, NT, and hypertensive (HT) eye retinas (Fig. 1b). First, we observed that RGC density differed between naïve and HT eyes. Indeed, the HT group exhibited a significant 22 % decrease (*p* = 0.0184) in RGC density in peripheral retina compared to the naïve group (Fig. 1c). Furthermore, we did not observe any loss of RGC in the middle and central retina in HT eyes compared with naïve eyes (data not show). Moreover, there was a trend to the decrease in RGC density in the NT group. Indeed, we found an 8 % non-significant decrease compared to the naïve group. Ocular hypertension induced RGC degeneration in peripheral retina 6 weeks after cauterization.

### Ocular hypertension induces retinal inflammation and tissue macrophage activation in HT and NT eyes

An earlier study showed that in an unilateral laser-induced OHT, the NT eye is also altered [20]. We aimed to explore and further detail this new data in our experimental model of OHT. In all experiments, we compared the EVC and contralateral structures to the naïve structures.

**Fig. 2:**
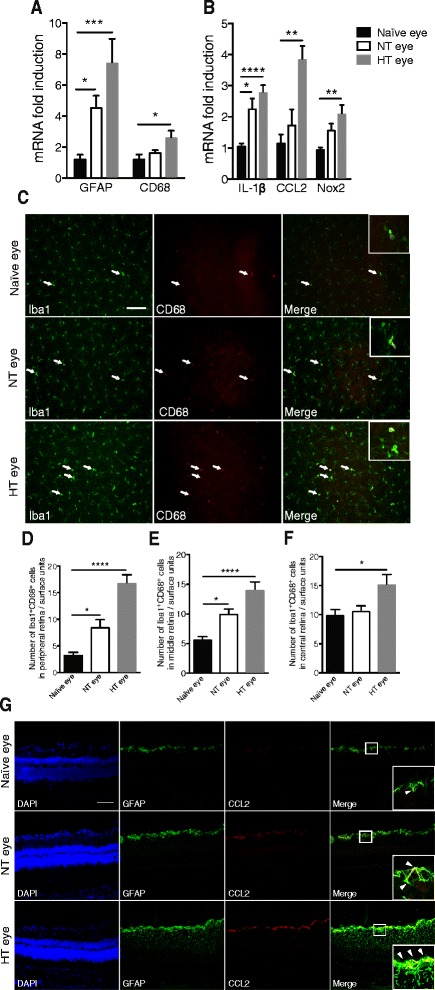
EVC induces retinal inflammation and microglial activation in both HT and NT eyes. **a**, **b** RT-qPCR analysis of the effect of cauterization on GFAP, CD68, IL-1β, CCL2, and Nox2 mRNA levels in naïve, NT, and HT eye retinas. mRNA levels are presented as a fold change relative to naïve rats after normalization with respect to the housekeeping gene (GAPDH). Each bar is the mean ± SEM. *n* = 8–10 animals/group. **c** Peripheral images from whole flat-mounted retina double-immunolabeled with Iba1 and CD68 antibody in naïve, NT, and HT eyes. *Arrows* show colocalization between markers. Scale bar = 100 μm. **c**–**e** Quantification of peripheral (**d**), middle (**e**), and central (**f**) mononuclear phagocyte (Iba1^+^CD68^+^ cells) density per retina in the naïve (*n* = 6), NT (*n* = 7), and HT (*n* = 7) eye groups. The average mononuclear phagocyte density values were determined from eight peripheral, middle, or central images per retina. **g** Confocal images of double-immunolabeling of CCL2 with GFAP, an astrocytic marker, in naïve, NT, and HT eye retinas. *Arrowheads* in inset show colocalization between markers. Scale bar = 100 μm. Results are expressed in arbitrary units and correspond to the means ± SEM. We performed a one-way ANOVA followed by the Kruskal–Wallis multiple comparisons post hoc test: *p < 0.05, **p < 0.01, ***p < 0.001, and ****p < 0.0001

EVC resulted in astrocyte activation (GFAP, *p* = 0.0007 with respect to naïve rats), in tissue macrophage activation (CD68, *p* = 0.0309 with respect to naïve rats) (Fig. 2a). There were also higher levels of mRNA for several key proinflammatory and oxidative stress factors: IL-1β (*p* < 0.0001), CCL2 (*p* = 0.0015), and Nox2 (*p* = 0.0023) in HT eye (Fig. 2b). For some genes, we found an increase in mRNA levels in the NT eye: GFAP (*p* = 0.0150) and IL-1 (*p* = 0.0132).

We found tissue macrophage activation in the HT eye retina and a trend toward an increase in the NT eye retina. To investigate this increase in mRNA levels in CD68 due to tissue macrophage/microglia activation, whole flat-mounted retina double immunohistofluorescence of Iba1 and CD68 was performed. Pictures represent peripheral areas of naïve, contralateral, and EVC retinas (Fig. 2c). We observed a change of microglia morphology in HT and NT eye retinas, showing that microglia cells acquire a reactive profile with short or even absent processes and large soma called ameboid.

Quantification of Iba1^+^CD68^+^ cells (tissue macrophages/microglia) in peripheral retina showed a twofold increase in contralateral eyes compared to naïve eyes and in HT eyes compared to NT eyes (Fig. 2d). We observed the same type of result in the middle retina but to a lesser extent (Fig. 2e). In the naïve eyes, the basal level of tissue macrophages/microglia appeared to be higher in the central retina than in the peripheral and middle retina. A significant increase was still found in the NT eyes compared to naïve eyes (Fig. 2f).

We also found a significant increase in the CCL2 mRNA level and in the number of tissue macrophages in HT eye retinas. Given that previous studies have assumed that CCL2 is strongly implicated in monocyte recruitment during inflammation [30], we further wished to determine which cells were expressing CCL2 in the present model.

### Ocular hypertension induces greater CCL2 expression by astrocytes in HT and NT eye retinas

We showed that OHT resulted in increased expression of CCL2 in HT eye retinas compared to naïve and NT eye retinas and in NT eye retinas compared to naïve retinas.

To define in which type of cells CCL2 was expressed, we double stained the retinas with CCL2 and the specific marker of astrocyte and Müller cell GFAP. Confocal images showed that CCL2 was widely co-localized with these cells specifically in the RGC layer (Fig. 2g). Moreover, GFAP and CCL2 labeling revealed a significant increase in HT eye retina compared to naïve retina. These data suggest that CCL2 is probably released by astrocytes and Müller cells in both basal and injury conditions.

### Retinal astrogliosis and microgliosis are transmitted along the ON

**Fig. 3:**
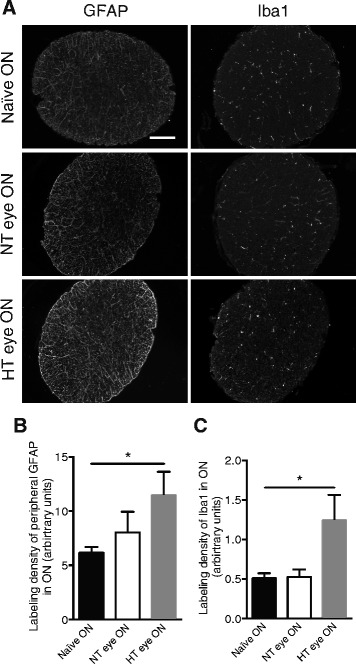
EVC induces astrogliosis and microgliosis in ON. **a** Immunofluorescent labeling for GFAP and Iba1 in naïve, NT, and HT eye ON. Scale bar = 100 μm. Quantification of GFAP (**b**) and Iba1 (**c**) immunofluorescence levels in naïve (*n* = 6), NT (*n* = 7), and HT (*n* = 7) eye ON. Results are expressed in arbitrary units and correspond to the means ± SEM. We performed a one-way ANOVA followed by the Dunnett multiple comparisons post hoc test: *p < 0.05

Segments of the ONs were examined for GFAP and Iba1 expression by immunofluorescence (Fig. 3a). Quantitative analysis of the surface area covered by GFAP staining within the ON revealed almost a twofold increase in the HT eye group compared to the naïve eye group (*p* = 0.0415) (Fig. 3b). Surprisingly, the extent of Iba1 immunoreactivity in the NT eye group was at the same level as in the naïve eye group but showed a twofold increase in the HT eye group (*p* = 0.0476) (Fig. 3c).

### Neuroinflammation in central visual projection pathways

**Fig. 4:**
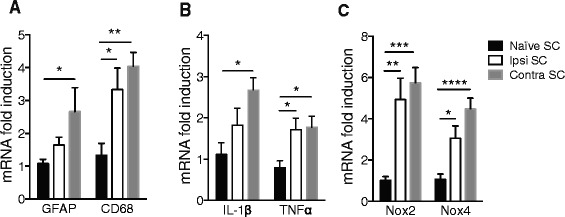
EVC induces an increase of inflammatory markers at mRNA levels in right and contralateral SC. RT-qPCR analysis for GFAP, CD68 (**a**), IL-1β, TNFα (**b**), Nox2, and Nox4 (**c**) mRNA levels in naïve, right, and contralateral SCs. For each marker, mRNA levels are presented as a fold change relative to naïve rats after normalization with respect to the housekeeping gene (GAPDH). Each bar is the mean ± SEM. *n* = 8–10 animals/group. We performed a one-way ANOVA followed by the Dunnett multiple comparisons post hoc test for GFAP, IL-1β, Nox2, and Nox4 and the Kruskal–Wallis test for TNF-α and CD68: *p < 0.05, **p < 0.01, ***p < 0.001, and ****p < 0.0001

After decussating, ON fibers divide in two ways in the rodent brain. The minor synaptic relay that concerns only a small percentage of fibers is the LGN. The major ON fiber relay in the rat brain is the SC. We found in the SC a similar profile of inflammation than in the retina (Fig. 4). EVC led to astrocyte and tissue macrophage activation (increase in GFAP and CD68 mRNA levels [*p* = 0.0136 and *p* = 0.0018, respectively], with respect to naïve rats) (Fig. 4a). There were higher levels of mRNA for proinflammatory and oxidative stress factors: IL-1β (*p* = 0.0115), TNFα (*p* = 0.0134) (Fig. 4b), Nox2 (*p* = 0.0004), and Nox4 (*p* < 0.0001) (Fig. 4c) in HT eye retinas. For some genes, we found an increase in mRNA levels in the NT eye: TNFα (*p* = 0.0175), CD68 (*p* = 0.0291), Nox2 (*p* = 0.003), and Nox4 (*p* = 0.0105).

**Fig. 5:**
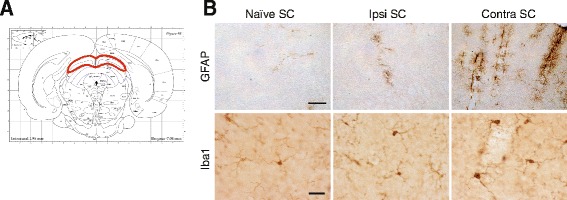
EVC induces astrogliosis and microgliosis in right and contralateral SCs. **a** Representative image of rat brain section showing the SC (*circled in red*). **b** GFAP and Iba1 staining in naïve, right (Ipsi), and contralateral SCs. Scale bar = 100 μm (GFAP) and 50 μm (Iba1)

To confirm these results, we performed immunochemistry in SCs. On the images of rat brain sections, SCs are circled in red (Fig. 5a). In naïve SCs, GFAP labeling was very rare and focused around the blood vessels and under the pia mater (Fig. 5b). In left SC, dense GFAP labeling was observed, indicating astrocyte activation. In left SCs, microglia seemed to have a wider soma than in naïve SCs (Fig. 5b).

### Ocular hypertension induces c-fos and phosphorylated/activated p38 (p-p38) activation in SC

**Fig. 6:**
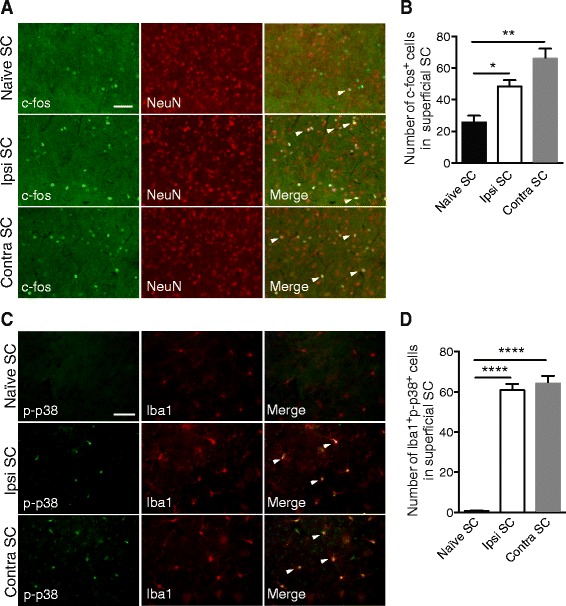
EVC induces inflammation, c-fos, and p-p38 pathway activation in right and contralateral SCs. **a** Immunofluorescence of c-fos and NeuN in naïve, right, and contralateral SCs. **b** Quantification of c-fos^+^ cells in naïve (*n* = 6), right (*n* = 7), and contralateral (*n* = 7) in superficial SCs. **c** Double-immunofluorescent labeling p-p38/Iba1 in naïve, right (Ipsi), and contralateral SCs. **d** Quantification of Iba1^+^p-p38^+^ cells in naïve (*n* = 6), right (*n* = 6), and contralateral (*n* = 6) SCs. Scale bars = 200 μm. Results are expressed in arbitrary units and correspond to the means ± SEM. We performed a one-way ANOVA followed by the Dunnett multiple comparisons test: *p < 0.05, **p < 0.01, ***p < 0.001, and ****p < 0.0001

Naïve, right and left SC sections were analyzed with immunofluorescence with c-fos and NeuN markers (Fig. 6a), and the number of neurons exhibiting the neuronal activation c-fos marker was counted (Fig. 6b). The quantitative analysis showed a twofold increase in the right group compared to the naïve group (*p* = 0.0385) and nearly a threefold increase in the left group compared to the naïve group (*p* = 0.001). These data suggested that retinal inflammation increased second-order neuron activation in right and left SCs compared to naïve SCs.

In the next series of experiments, we investigated the activation of the p-p38 pathway in the SCs (Fig. 6c). In naïve SCs, only a few cells were immune-positive for p-p38. In contrast, in right and left SCs, p-p38 was exclusively detected in microglia and the positive cell distribution did not change between right and left SCs. Quantification of p-p38-positive cells confirmed that this activation only occurred in right and left SCs (Fig. 6d) (*p* < 0.0001).

### HT eye inflammation is mostly transmitted to the NT eye via the SCs

**Fig. 7:**
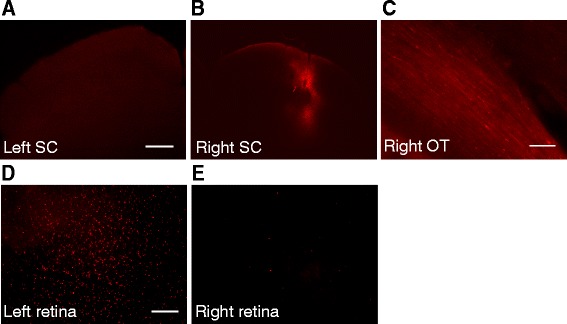
Unilateral CTB injection in the right SC labels RGCs in both retinas. **a** CTB Alexa 594 was stereotaxically injected into the right SC. No staining was observed in the left SC. **b** CTB in the right SC follows the needle path. Scale bar = 400 μm. **c** Neuronal fibers containing the CTB tracer in the right optic tract (OT). Scale bar = 100 μm. **d** Numerous RGCs labeled with CTB in the left retina. **e** Only a few RGCs are labeled by CTB in the right retina. Scale bar = 200 μm

We hypothesized that the SC (the first relay in the rat visual system) is the structure by which inflammation can be transmitted to the contralateral eye. To verify this hypothesis, we injected the CTB Alexa 594 into the right SC of naïve rats. First, to test the quality of injection, we made a single injection of CTB in the right SC (Fig. 7b). We did not find CTB in the left SC (Fig. 7a). Then the injection was localized in the superficial layers of the SC [31], with most of the CTB fluorescence (in red) located in the superficial SC (Fig. 7b). Five days after injection in the right SC, the CTB was found in the right optic tract nerve fibers (Fig. 7c). In rats, the majority of fibers originating from the left retina projects to the right SC. Indeed, we found a large number of RGCs containing the fluorescent tracer in the left retina (Fig. 7d), while in the right retina less stained RGCs were detected (Fig. 7e).

**Fig. 8:**
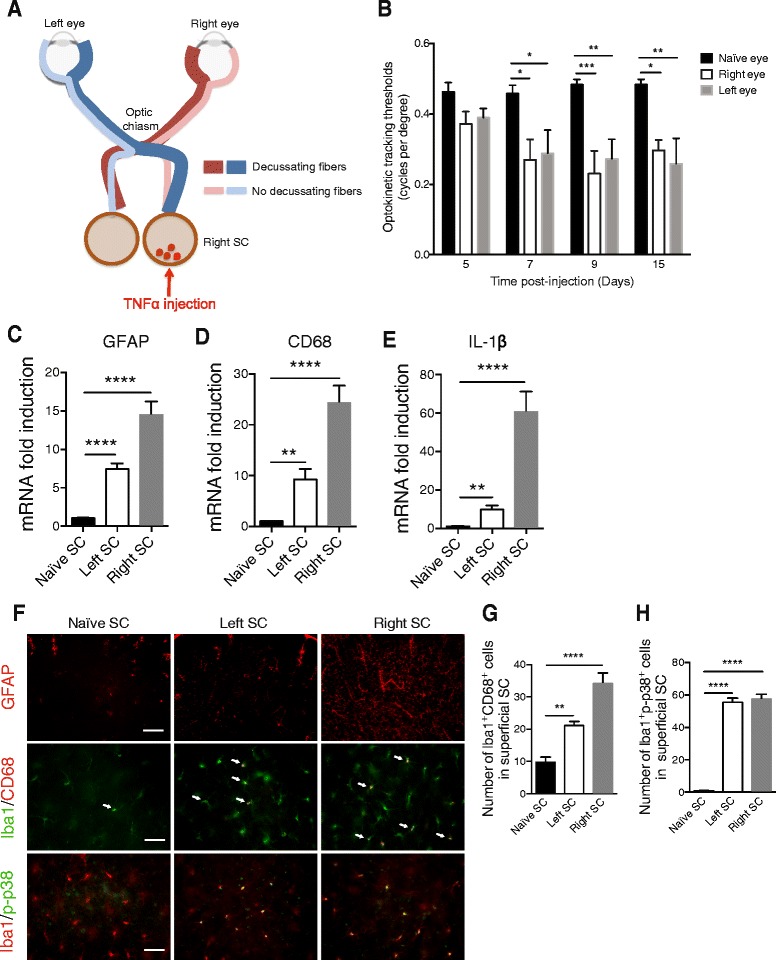
Unilateral TNFα injection induces visual defect, inflammation, and p-p38 pathway activation in both SCs. **a** Diagram showing the neuronal visual pathway in rodent. TNFα (25 ng/μl) is injected into the right SC. **b** The optomotor set-up allowed the determination of the optokinetic tracking threshold measurements (cycles per degree) for the left and right eyes, independently scored (clockwise and counterclockwise responses, respectively) under photopic conditions. Right and left eye sensitivity for naïve (*n* = 5), left (*n* = 6), and right (*n* = 6) rats at 5, 7, 9, and 15 days after TNFα injection. RT-qPCR analysis for GFAP (**c**), CD68 (**d**), and IL-1β (**e**) mRNA levels in naïve, left, and right SCs. For each marker, mRNA levels are presented as a fold change relative to naïve rats after normalization with respect to the housekeeping gene (GAPDH). Each bar is the mean ± SEM. *n* = 8–10 animals/group. Immunofluorescence of GFAP, Iba1/CD68, and Iba1/p-p38 (**f**) in naïve, left, and right SCs. *Arrows* show colocalization between markers. Scale bar = 200 μm (GFAP) and 50 μm (Iba1/CD68 and Iba1/p-p38). Quantification of Iba1^+^CD68^+^ cells (**g**) and Iba1 + p-p38+ cells (**h**) in naïve (*n* = 5), left (*n* = 6), and right (*n* = 6) SCs. Results are expressed in arbitrary units and correspond to the means ± SEM. Two-way ANOVA for repeated measures followed by Bonferroni post hoc test was used for optomotor responses: *p < 0.05, **p < 0.01, and ***p < 0.001 naïve, right, and left eyes. We performed a one-way ANOVA followed by the Dunnett multiple comparisons test: **p < 0.01, ***p < 0.001, and ****p < 0.0001 and the Kruskal–Wallis test for IL-1β

After validating the quality and depth of injection, we further injected TNFα (25 ng/μl) into the right SC of naïve rats. Figure 8a illustrates the rat visual pathway and the injection site. We observed whether or not RGC fibers decussate at the optic chiasma. We also assessed whether the treatment could impair rat visual function by studying their optomotor responses to rotating sinusoidal gratings 5, 7, 9, and 15 days after TNFα injection into the SC (Fig. 8b). These data indicate that the visual function of rats treated with TNFα was impaired from 7 days up to 15 days after injection (*p* = 0.0446 for the left eye versus the naïve eye and *p* = 0.0211 for the right eye versus the naïve eye at 7 days; *p* = 0.0032 for the left eye versus the naïve eye and *p* = 0.0006 for the right eye versus the naïve eye at 9 days; *p* = 0.0025 for the left eye versus the naïve eye and *p* = 0.0146 for the right eye versus the naïve eye at 15 days).

RT-qPCR analysis of pro-inflammatory genes confirmed our previous results. We observed a significant increase in GFAP mRNA for astrogliosis (Fig. 8c) (*p* < 0.0001) in CD68 mRNA for tissue macrophage/microglia activation (Fig. 8d) (*p* < 0.0001) and in IL-1β mRNA (Fig. 8e) (*p* < 0.0001) in the right SC compared to a naïve SC. Some genes were increased in the left SC, such as GFAP (*p* < 0.0001), CD68 (*p* = 0.0044), and IL-1β (*p* = 0.0090).

TNFα injection produced wide astrogliosis and tissue macrophage/microglia activation (Fig. 8f) in both left and right SCs. Quantification of the number of Iba1^+^CD68^+^ cells showed an increase in the number of tissue macrophages/microglia in the left and right superficial SCs compared to a naïve SCs (Fig. 8g). We also observed an activation of the p-p38 pathway in microglia in the left and right SCs, while no p-p38-positive cells were seen in naïve SCs (Fig. 8f). Quantification of the number of Iba1^+^p-p38^+^ cells confirmed these results (Fig. 8h).

**Fig. 9:**
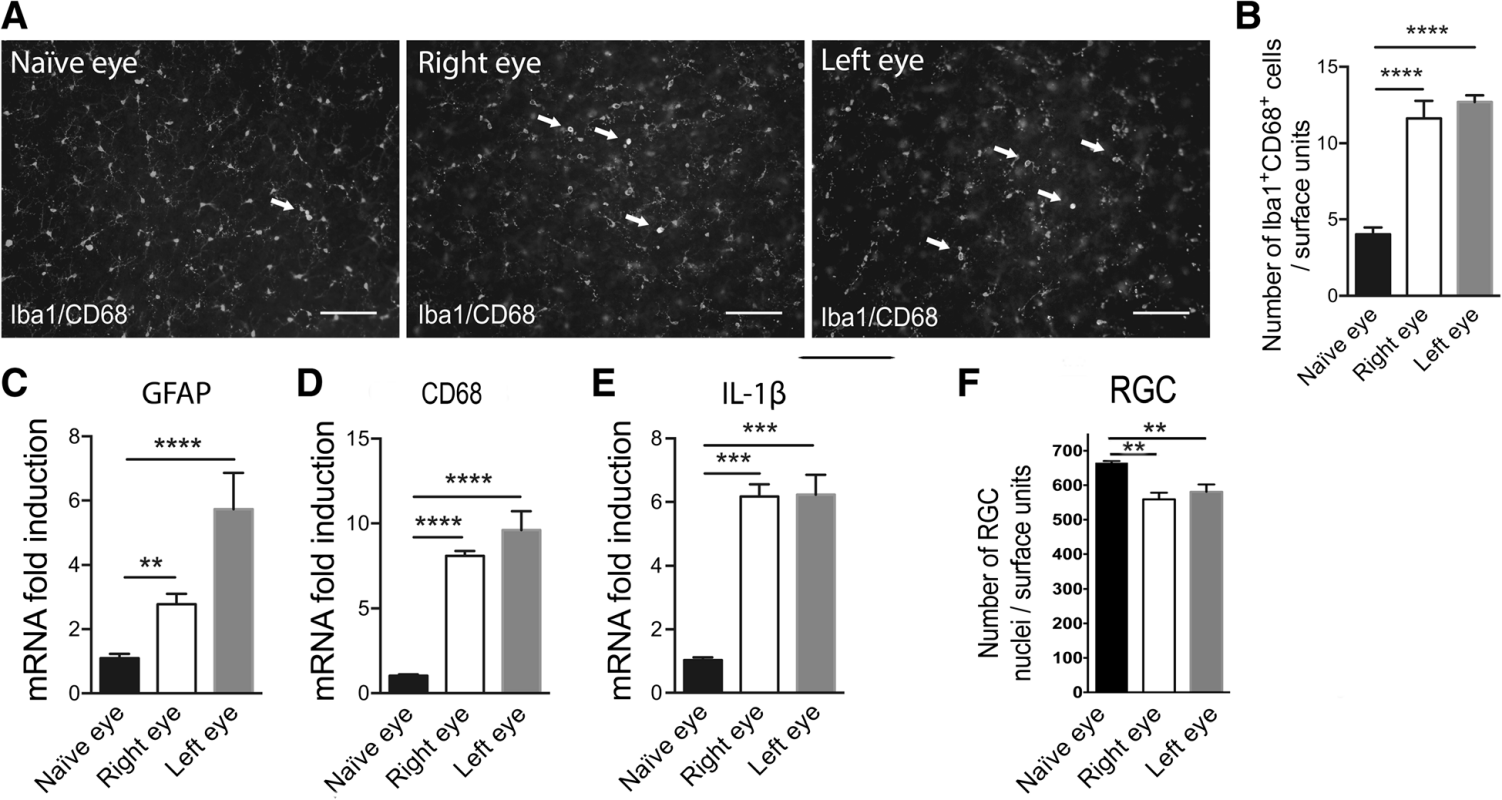
SC inflammation induces inflammation and RGC loss in both retinas. **a** Whole flat-mounted retina double-immunolabeled with Iba1 and CD68 in naïve, right, and left eyes of TNFα-injected rats. *Arrows* show colocalization between markers. Scale bar = 100 μm. **b** Quantification of Iba1 + CD68+ cells per retina in the naïve group (*n* = 6), right eye (*n* = 6), and left eye (*n* = 6) groups. RT-qPCR analysis for GFAP (**c**), CD68 (**d**), and IL-1β (**e**) mRNA levels in naïve, left, and right retinas. For each marker, mRNA levels are presented as a fold change relative to naïve rats after normalization with respect to the housekeeping gene (GAPDH). Each bar is the mean ± SEM. *n* = 8–10 animals/group. Results are expressed in arbitrary units and correspond to the means ± SEM. **f** Quantitation of RGCs loss in the projecting retina after TNFα stereotaxic microinjection into the right SC. The resulting values were expressed in surface units corresponding to 306,804 μm^2^. We performed a one-way ANOVA followed by the Dunnett multiple comparisons test: **p < 0.01, ***p < 0.001, and ****p < 0.0001 and the Kruskal–Wallis test for GFAP, and IL-1β

To evidence damage in both eyes, we performed whole flat-mounted retina double immunohistochemistry for Iba1 and CD68. The images show areas of naïve, right and left retinas (Fig. 9a). We observed morphological changes of microglia in the right and left retinas corresponding to activated tissue macrophages. Quantification of Iba1^+^CD68^+^ cells (tissue macrophage/microglia activation) in retinas showed more than a twofold increase in right and left eyes compared to naïve eyes (Fig. 9b). To support these results, we performed RT-qPCR analysis in the left and right retinas in TNFα-treated rats compared to naïve rats. We observed a significant increase of GFAP (Fig. 9c) (*p* < 0.0001 in the left retina and *p* = 0.0074 in the right retina compared to a naïve retina), CD68 (Fig. 9d) (*p* < 0.0001 in the left retina and *p* < 0.0001 in the right retina compared to a naïve retina), and IL-1β (Fig. 9e) (*p* = 0.0006 in the left retina and *p* = 0.0005 in the right retina compared to a naïve retina) mRNA in both retinas. Then we evaluated RGC density in the projecting retina after TNFα microinjection into the right SC (Fig. 9f). We observed that TNFα injected into the right SC induces a loss of ≈20 % of RGC in both retinas as compare to control (*p* < 0.001 in the left retina and *p* < 0.001 in the right retina compared to a naïve retina).

## Discussion

In this study, we used an experimental model of glaucoma (induced by EVC), which is very close to the human form of the disease. Numerous studies have characterized and used this EVC model, which matches specific glaucoma symptoms: IOP increase with a reduction of aqueous humor outflow, progressive RGC degeneration, aqueous humor TGF-β2 level increase, and trabecular cell death [2, 3, 32]. The EVC model used in this study shares several features with human primary open-angle glaucoma, but it is known there is blood flow damage. Indeed, following episcleral/vortex veins cauterization, arterial blood is suddenly left with insufficient outflow from the globe. This would rapidly produce significant ocular venous congestion, which is consistent with an immediate rise in IOP observed in this ocular hypertensive model [33]. In this EVC glaucoma model, RGC loss is progressive (22 % decrease in peripheral retina 6 weeks after cauterization). This peripheral RGC loss has also been observed in different glaucoma models with, however, a markedly different percentage of RGC loss. One study reports an 11 % decrease of RGC density 12 weeks after intracameral microbead injection in mice [34], while another group showed a 30 % decrease 4 months after an association of microbead injection and laser photocoagulation of the trabeculum meshwork in mice [35]. Other groups have developed EVC model but in other rat strains. Thus, one study reported 15 % of RGC loss in peripheral retina 8 weeks after EVC. In another study, the authors found a decrease of 40 % in RGC density 26 weeks after EVC [36], whereas two other groups observed, 7 weeks after EVC, 30 % of RGC loss in peripheral retina [37] and in the whole of the retina for the other [38], respectively.

In neurodegenerative disease, a neuronal degeneration is almost always associated with inflammation. Therefore, in glaucomatous human eyes, an activated phenotype of astrocytes, Müller cells and microglia cells are found in retinal layers [39]. Indeed, the hallmarks of glial and microglial activation such as cellular body hypertrophy and increased expression of glial intermediate filaments (GFAP) and tissue macrophage/microglia marker such as CD68 are classically observed in glaucomatous eyes in humans [40] as well as in experimental models of OHT [11, 41, 42].

In this study, we demonstrated that the increase of the number of activated tissue macrophages/microglia in the HT eye retina is correlated with an increase in CCL2 mRNA. In addition, we observed that CCL2 chemokine is expressed by astrocytes. It is well known that CCL2 is strongly implicated in monocyte chemoattractivity from blood circulation to the inflammatory site [30]. Activated tissue macrophages could stem from either activation of resident microglia or activation of infiltrating monocytes. Tissue macrophage/microglia activation could be responsible for the increase in pro-inflammatory cytokines (TNFα and IL-1β) observed in retina [42]. This result is corroborated with a recent study that clearly demonstrated that pro-inflammatory mediators signal the progression of pathological changes in visual projection in a DBA/2J glaucoma model prior to functional transport loss and RGC death [18].

We hypothesized that loss of RGCs can lead to RGC axon impairments and ON gliosis. In this way, we clearly observed a strong radial astrogliosis and tissue macrophage activation in the ON of the HT eye. These results are consistent with other studies that have examined glaucomatous ON. In a diode laser burn model, strong GFAP immunostaining was found associated with a degeneration of ON fibers [43]. In a 12-month-old DBA/2J model, the authors are found lower β-Tubulin labeling than in the control mice [44]. This suggests an anterograde deficit transport that can lead to damage in SCs. In our experimental model of glaucoma, the SC is also injured. The left SCs showed astrogliosis, microglia activation, and elevated pro-inflammatory cytokine expression. In a model of acute perfusion of the anterior chamber of the right eye with saline solution, they found an increase in the number of GFAP-positive astrocytes throughout the superficial layers of the left SC [17]. In a model of laser photocoagulation of the perilimbal and episcleral veins, they found a significant enlargement of the GFAP within the left SC and also in the right SC [45].

Increasing evidence supports that the glaucomatous tissue stress (apoptosis of RGCs) initiated by inflammation involves reactive oxidative species (ROS) [46, 47]. This study found an increase in an oxidative stress marker (Nox2) in HT eye retina. The Nox2 enzyme is implicated in ROS synthesis, particularly in superoxide ion production. This result is corroborated by the demonstration that in another experimental model in which glaucoma is induced by chronic injection of hyaluronic acid in the eye’s anterior chamber, a decrease in superoxide dismutase and catalase activity was observed in total retina after 1 month [48]. Moreover, we found that oxidative stress markers, Nox2 and Nox4, were also significantly elevated in SCs. It is already known that oxidative stress by ROS overproduction may significantly contribute to neurodegeneration [49].

In the present study, we hypothesized that retinal inflammation in the HT eye could be transmitted to the NT eye via the SC (the main projection site of RGCs in rodents). Indeed, RGC axons from one retina project to both SCs with 10–15 % RGC fibers coming from the right eye and 85–90 % from the contralateral eye [14–16, 22, 23, 25]. In our unilateral glaucoma model, we observed an activation of second-order neurons and/or interneurons in both SCs. Indeed, there is a significant increase in the number of c-fos-positive cells in both SCs compared to naïve SCs. c-fos is a marker that could identify activated neurons in histological preparations. c-fos is a proto-oncogene that is expressed within neurons following voltage-gated calcium entry into the cell [50]. Neuronal excitation leads to a rapid and transient induction of c-fos. We demonstrated that c-fos is activated in left and right SC after unilateral OHT. We postulated that neuroinflammation and RGC death induce activation of the second-order neurons in both SCs. The right SC analysis revealed, in an original way, an injury characterized by microglia activation and elevated pro-inflammatory cytokine expression. We hypothesized that the 10–15 % of RGC fibers from the HT eye induce neuroinflammation in the right SC.

We also found activation of the p-p38 pathway in microglia in both left and right SC. It is already known that p-p38 immunostaining was found in glia of human glaucomatous retina [39]. Moreover, p38 could be activated and phosphorylated by pro-inflammatory cytokines such as TNFα and IL-1β [51]. This observation is particularly true in our model because we found an increase in TNFα expression in both left and right SC. p-p38 activation is also involved in apoptotic signaling, especially in RGCs [52]. The NT eye analysis of EVC rats also displayed astrogliosis and tissue macrophage/microglia activation. A research group has already shown damage in the NT eye but only in the retina of OHT animals [19, 20]. We wished to identify how this inflammation could be transmitted to the NT eye. In the present experimental model of OHT, the results clearly show that the widespread inflammation observed in the HT eye spread to the NT eye. We hypothesized that the SC is the major site of antero- and retrograde communications between the eyes. To test this hypothesis, we injected an inflammatory cytokine (TNFα) into the right SC, which receives approximately 10–15 % RGC fibers from the right eye and 85–90 % RGC fibers from the left eye. Thus, we showed that after unilateral injection of TNFα into the right SC, both right and left SCs present astrogliosis and tissue macrophage/microglia activation. In addition, a significant increase in the number of activated tissue macrophages in both left and right eyes was associated with inflammation. These results contribute new information to the history of glaucoma development. Inflammation in one eye could activate RGC fibers and induce inflammation in both SCs, which in turn may activate inflammation in the other eye by retrograde transport by way of RGC fibers. To corroborate this hypothesis, RGCs have been traced from the SC using a neurotracerdye such as Fluorogold [53], which was preferentially concentrated in the cell body of RGCs during the next 3–4 days. Moreover, in a model of Parkinson disease, a retrograde degeneration of dopaminergic neurons was observed after 6-hydroxydopamine (6-OHDA) injection in the striatum, which receives substantia nigra dopaminergic neuronal fibers. Striatal fibers degenerate, followed by cellular bodies located in substantia nigra [54].

## Conclusions

These results demonstrate that elevated IOP induces neuroinflammation in the retina of the damaged eye but not only at the RGC level. This inflammation is strongly associated with RGC degeneration and leads to stress signal transmission in both SCs by RGC axons. We observed glial cell activation in both SCs and it is well known that activated glial cells produce a wide range of inflammatory molecules that could alter neuronal functioning. Damaged neurons of SCs will in turn induce neuroinflammation in the left retina that contains RGCs cell bodies and whose RGC fibers project in both the left and right SCs. All of these data evidence the complex role of the SCs in the propagation of neuroinflammatory events induced by glaucoma and provide new insight onto the development of neurodegenerative diseases such as glaucoma.

## Abbreviations

CTB: cholera toxin subunit B
EVC: episcleral vein cauterization
HT: hypertensive
IOP: intraocular pressure
LGN: lateral geniculate nucleus
NT: normotensive
OHT: ocular hypertension
ON: optic nerve
RGC: retinal ganglion cell
SC: superior colliculus
